# Agritourism and Kidding Season: A Large Outbreak of Human Shiga Toxin-Producing *Escherichia coli* O157 (STEC O157) Infections Linked to a Goat Dairy Farm—Connecticut, 2016

**DOI:** 10.3389/fvets.2021.744055

**Published:** 2021-11-16

**Authors:** Megin C. Nichols, Paul Gacek, Quyen Phan, Kelly J. Gambino-Shirley, Lauren M. Gollarza, Morgan N. Schroeder, Alexandra Mercante, Jocelyn Mullins, Anna Blackstock, Mark E. Laughlin, Samantha M. Olson, Eugene Pizzo, Tu Ngoc Nguyen, Laurn Mank, Kimberly Holmes-Talbot, Alycia McNutt, Diane Noel, Anthony Muyombwe, Jafar H. Razeq, Mary Jane Lis, Bruce Sherman, Wayne Kasacek, Laura Whitlock, Nancy Strockbine, Haley Martin, Eshaw Vidyaprakash, Patrick McCormack, Matthew Cartter

**Affiliations:** ^1^Division of Foodborne, Waterborne and Environmental Diseases, National Center for Emerging and Zoonotic Infectious Diseases, Centers for Disease Control and Prevention, Atlanta, GA, United States; ^2^Connecticut Department of Health, Hartford, CT, United States; ^3^Oak Ridge Institute for Science and Education (ORISE), Oak Ridge, TN, United States; ^4^Connecticut Department of Agriculture, Hartford, CT, United States; ^5^Uncas Health District, Norwich, CT, United States

**Keywords:** *E. coli*–*Escherichia coli*, goat, outbreak, agritourism, diarrhea, Shiga toxin (Stx) producing *Escherichia coli* (STEC), children, hemolytic uremic syndrome

## Abstract

The objective of this study was to determine sources of Shiga toxin-producing *Escherichia coli* O157 (STEC O157) infection among visitors to Farm X and develop public health recommendations. A case-control study was conducted. Case-patients were defined as the first ill child (aged <18 years) in the household with laboratory-confirmed STEC O157, or physician-diagnosed hemolytic uremic syndrome with laboratory confirmation by serology, who visited Farm X in the 10 days prior to illness. Controls were selected from Farm X visitors aged <18 years, without symptoms during the same time period as case-patients. Environment and animal fecal samples collected from Farm X were cultured; isolates from Farm X were compared with patient isolates using whole genome sequencing (WGS). Case-patients were more likely than controls to have sat on hay bales at the doe barn (adjusted odds ratio: 4.55; 95% confidence interval: 1.41–16.13). No handwashing stations were available; limited hand sanitizer was provided. Overall, 37% (29 of 78) of animal and environmental samples collected were positive for STEC*;* of these, 62% (18 of 29) yielded STEC O157 highly related by WGS to patient isolates. STEC O157 environmental contamination and fecal shedding by goats at Farm X was extensive. Farms should provide handwashing stations with soap, running water, and disposable towels. Access to animal areas, including animal pens and enclosures, should be limited for young children who are at risk for severe outcomes from STEC O157 infection. National recommendations should be adopted to reduce disease transmission.

## Introduction

*Escherichia coli* O157:H7 causes an estimated 95,000 illnesses and 30 deaths annually in the United States (1, 2). Zoonotic transmission of Shiga-toxin producing *Escherichia coli* (STEC) causes an estimated 5,960 annual US infections (3). In Connecticut, there are an average of 23 STEC illnesses annually; 113 total illnesses were reported during 2011–2015 (4). Some people with STEC infections, particularly young children, can develop hemolytic uremic syndrome (HUS), a severe complication that can lead to extended hospitalizations, kidney failure, thrombocytopenia, and death (5, 6). Non-specific supportive therapy, including hydration, is important in treating STEC infections (5). Antibiotics are not recommended to treat infections, since there is no evidence that treatment with antibiotics is helpful and taking antibiotics might increase the risk of HUS (6, 7). Antidiarrheal agents might also increase that risk (8).

STEC O157 bacteria are often transmitted through the fecal-oral route, including from contact with animals or the environment. As with many enteric (intestinal) pathogens, STEC O157 can persist in contaminated environments for long periods of time (up to 10 months) (9, 10). During 1991 to 2005, the CDC received reports of 32 outbreaks of STEC O157 associated with animals in public settings in the United States (11). To address this, the National Association of State Public Health Veterinarians (NASPHV) created the Compendium of Measures to Prevent Disease Associated with Animals in Public Settings in 2005 (Compendium) (12). In its latest version published in 2017, the Compendium described recommendations for minimizing health risks associated with animal contact in public settings (13). The Compendium encourages appropriate risk reduction and careful management of animals that have contact with the public.

Healthy ruminants are a known reservoir of STEC O157. Although studies often cite cattle as the primary source of STEC O157 outbreaks in humans, recent epidemiological studies have turned attention to sheep and goats as equally significant sources of STEC O157 human infection (14–21). The increasing popularity of petting farms and zoos and agritourism on working farms creates greater potential for human interaction with goats and their environments. Therefore, in addition to standard public health recommendations, finding ways to decrease shedding of STEC O157 into the environment by goats might help prevent human illness (19).

Contact with goats and their environment (such as areas where they live and roam) has led to numerous STEC O157 infection outbreaks in humans (15, 16, 18, 21). In 2004, an outbreak of STEC O157 infections affected 108 visitors to the North Carolina State Fair petting zoo, including 15 who developed HUS (22). Visitors at this fair could interact directly with ~100 goats and sheep. Risk factors in this outbreak included touching or stepping in manure or sitting on the ground near animal bedding (22). STEC O157 was isolated from the bedding 10 days after the fair had ended and from soil 5 months after the bedding and topsoil had been removed from the premises (22). In 2005, an outbreak of STEC O157 infections affected 63 people who had contact with goats at multiple fairs in Florida; of these, 7 developed HUS (22). In this outbreak, illness was most strongly associated with direct animal contact or contact with sawdust or shavings. In 2007, an outbreak of STEC O157 infections was associated with a goat at a day camp petting zoo in Florida. Signage and numerous handwashing stations at the petting zoo decreased disease risk, but human illnesses still occurred (15). In each of these outbreaks linked to goats, investigators concluded prevention measures focusing on improving infection control measures and avoiding risk behaviors were needed at fairs and other animal exhibits to further reduce disease transmission and prevent similar outbreaks.

Agritourism in the United States has increased in recent years. In 2012, 33,161 farms in the United States earned over $704,038,000 from agritourism-related activities, representing an income increase of ~24% over 2007 (23). These farms offer public activities such as animal exhibitions, petting zoos, corn mazes, fruit picking, educational tours for children, farm-based festivals, country markets, and stores (24). Farms participating in agritourism are visited by millions of children each year (24). Many children and adults are new to the experience and unfamiliar with risks associated with agriculture environments (24, 25). These risks are described in journal articles and legal briefs which emphasize the responsibility of those managing animal exhibitions duty of care to invitees and visitors of the premises (e.g., farm, fair, ranch, zoo) (26, 27). Per these articles, failing to implement established recommendations of public health organizations, including education and adequate reduction of risks, might constitute falling below that standard of care and might be harmful to farm visitors and to the viability of the venue (16, 26).

On March 24, 2016, the Connecticut (CT) Department of Public Health (DPH) identified a cluster of seven culture-confirmed STEC infections; six of seven (86%) ill people reported visiting the same goat dairy farm in Southeastern CT (Farm X) in the week before illness onset. An investigation was initiated by the CT DPH, the CT Department of Agriculture (DoA), CDC, and the local health district to determine the extent of the outbreak, identify risk factors and potential sources of infection, and develop recommendations to prevent further illnesses.

## Materials and Methods

The CT DPH laboratory (DPHL) performed culture of samples and pulsed-field gel electrophoresis (PFGE) on the isolates using standard protocols (28). These data were shared with PulseNet, the national molecular subtyping network for foodborne disease surveillance, to characterize STEC O157 isolated from people, animals, or the farm environment during the outbreak investigation; the resultant STEC O157 isolates were used to define the outbreak strain. Whole genome DNA sequencing (WGS) was conducted by CT DPHL using PulseNet protocols on the Illumina MiSeq Sequencing System (29). High quality single nucleotide polymorphism (SNP) analyses were generated by PulseNet with Lyve-SET version 1.1.4f using an internal reference (*E. coli* O157: PNUSAE002903; *E. coli O103*: PNUSAE003156) with phages masked. Reads were cleaned with CG Pipeline (options: –no-singletons). Single nucleotide polymorphisms (SNPs) were called with Varscan, and Lyve-SET was run with the following options: minimum coverage −20, min alternative fraction −0.95, and allowed flanking −5 bp (30). All sequence data generated by PulseNet were deposited to the National Center for Biotechnology Information (NCBI) in Bioproject PRJNA218110. Serology was performed by the National Enteric Reference Lab at CDC to examine presence of antibodies in sera from patients with HUS against the O157 lipopolysaccharide antigen.

For investigation purposes a case was defined as laboratory-confirmed STEC O157 infection with the outbreak strain or physician diagnosis of HUS with laboratory confirmation by serology, during March 7, 2016, to May 14, 2016 residing in CT or surrounding states.

### Statistical Methods

To further examine risk factors for illness among children who visited Farm X, a case-control analysis was initiated and limited to case-patients and controls aged <18 years. For the case-control analysis, a case-patient was defined as the first ill child (aged <18 years) in a household with laboratory-confirmed STEC O157 or physician diagnosis of HUS with laboratory confirmation by serology who visited Farm X within the 10 days prior to illness onset. Controls were selected from households with children (aged <18 years) who contacted DPH as requested by press releases. Controls were required to be without any signs or symptoms of STEC O157 infection within the 10 days following their Farm X visit from March 7–May 14, 2016. Our attempted enrollment was at least two controls for every case-patient. Between April 5th and 21st, we administered a standard questionnaire by phone to obtain information regarding the visit to the farm, demographic information, risk factors including hand to mouth habits, activities at the goat barns, tasting samples, and hand sanitizer use. A parent was interviewed for case-patients and controls. Behavioral risk factors were assessed by asking the parent to report if their child performed that behavior at any point during the visit to Farm X. We fit univariable logistic regression models and calculated unadjusted and adjusted odds ratios and exact 95% confidence intervals (CI) (SAS, version 9.3, SAS Institute Inc, Cary, NC). A multivariable logistic regression model was performed to examine odds of illness after sitting on hay bales in the doe barn, adjusting for age <5 years and using hand sanitizer after visiting the kid barn. These variables were selected as they were thought to be risk factors for developing illness (age) or for preventing illness (hand sanitizer use). The strength of association of variables in relation to the outcome of infection was assessed using odds ratio point estimates and confidence intervals. As a result of small numbers, we used the Firth penalized likelihood for most models but used exact logistic regression for variables with extremely small numbers (<1). For the multivariable model, we included variables of interest based on previous studies.

Environmental sampling was conducted on the farm on March 28, 2016. CT DoA collected animal fecal samples with assistance from CDC on March 29, 2016. Environmental and animal sampling was conducted using a sterile cotton swab at various locations in the kid barn, doe barn, farm store, and farm grounds including the pens, barn floor, gutters, bedding, hay bales, rafters, troughs, walls inside pens, a grain holder, and muddy runoff from a compost pile. Does and goat kids were tested. Swabs were inserted into Cary Blair semisolid transport media. Feces, bedding, and grain samples were collected using stool cups and sterile tongue depressors. Two water samples were collected using sterile plastic bottles. All samples were transported on ice in secondary containers within a cooler to the CT DPHL. Initial setup of environmental samples at CT DPHL was performed following recommendations from the National Enteric Reference Lab at CDC. Swabs were received in Cary Blair transport media and were directly plated onto Sorbitol MacConkey Agar with Cefexime & Tellurite (CT-SMAC) and Sorbitol MacConkey Agar (SMAC) plates. Following plating, the swabs were placed into 8 ml of Gram Negative (GN) broth. All bedding, fecal, grain, and water samples were enriched at a 1:10 dilution in GN broth. Plates and broths were incubated at 37°C and examined for growth after 18 h. Plates and broths with no growth were re-incubated for an additional 18–24 h at 37°C. Plates and broths with growth were screened with the Meridian Premier Enterohemorrhagic (EHEC) *E. coli* Enzyme Immunoassay (EIA) for evidence of STEC. Individual sorbitol negative colonies from positive plates were screened with Remel RIM™ O157:H7 latex test kit. Remel RIM™ O157:H7 latex positive colonies were plated to Trypticase Soy Agar Plates with 5% Sheep Red Blood (BAP) for biochemical testing and submission to the PFGE Laboratory. EHEC EIA positive broths were plated to SMAC and CT- SMAC plates, plates were incubated at 37°C for 18 h and growth was screened for STEC with the Meridian Premier EHEC *E. coli* EIA. Individual sorbitol negative colonies from positive plates were screened with Remel RIM™ O157:H7 latex test kit. Remel RIM™ O157:H7 latex positive colonies were plated to BAP for biochemical testing and submission to the PFGE Laboratory.

Plates positive for STEC by the Meridian Premier EHEC *E. coli* EIA but negative for *E. coli* O157:H7 were screened for non-O157 STEC. Individual sorbitol positive colonies were screened by EHEC EIA. Positive colonies were plated to BAP for biochemical testing, serogrouping, and submission to the PFGE Laboratory. Samples were cultured at the CT DPHL; STEC isolates from Farm X were compared with patient isolates using PFGE and WGS. Non-O157 STEC colonies were serogrouped with the six most common O-antigens known to produce toxins (O26, O45, O103, O111, O121, and O145) by agglutination method. The owner of Farm X was interviewed regarding farm operations and sanitation practices. CT DoA inspected the milk pasteurization process on equipment located on Farm X and collected samples of cheeses and milk which were produced for sale to farm visitors. This investigation was reviewed and did not meet the definition of research under 45 CFR 46.102(d). IRB review was not required. This activity was reviewed by CDC and was conducted consistent with applicable federal law and CDC policy.

## Results

Fifty-one laboratory-confirmed cases were identified; of these, one had physician-diagnosed HUS and antibodies to the O157 lipopolysaccharide antigen by serology. The median age was 4 years (range: <1–50 years). Nine patients (18%) were adults and 43 (84%) were children <18 years of age; of these 29 (67%) were children ≤ 5 years of age. Twenty-eight patients (55%) were male. Eleven patients (22%) were hospitalized, and three (6%) developed HUS. No deaths occurred. Illness onset dates ranged from March 7 to May 14, 2016. All patients were interviewed. Forty-two patients (82%) visited Farm X or had contact with goats originating from the farm, seven (14%) had contact with someone who visited the farm, and three (6%) did not have any identified epidemiologic link to the farm or goats from the farm. One additional patient was an asymptomatic household contact of a symptomatic *E. coli* O157 patient. The asymptomatic patient, who did not visit the farm, was detected because household contacts of a STEC patient are recommended to be tested before returning to daycare in Connecticut.

The case-control analysis included 23 case-patients and 44 controls (Table 1). No evidence of a difference in age or sex was observed between case-patients and controls (Table 1). Risk factors, including hand to mouth habits, activities at the goat barns, tasting samples, and hand sanitizer use were analyzed (Table 1). Case-patients had higher unadjusted odds than controls for having sat on hay bales at the doe barn (odds ratio: 4.85; 95% CI: 1.54–16.40). In the multivariable analysis, the odds of being a case patient was 4.55 times higher for those that sat on hay bales (95% CI: 1.41–16.13, Table 2).

**Table 1:** Univariable analysis of other risk factors for STEC O157 infection among children visiting farm X—Connecticut, 2016 (*n* = 67).

**Exposure category**	**Case-patient** **(*n* = 23) ** **No. (%)**	**Control** **(*n* = 44)** **No. (%)**	**Odds ratio** **(95% confidence** **interval)**
Age^*^			
<5 years	14 (61)	22 (52)	1.39 (0.51–3.92)
5–17 years	9 (39)	20 (47)	Ref
Sex^*^			
Female	10 (43)	20 (47)	0.89 (0.32–2.42)
Immunosuppression	0 (0)	1 (2)	1.91 (<0.01–36.35)
Live on a property with farm animals	3 (13)	9 (20)	0.64 (0.15–2.29)
Multiple farm visits	3 (13)	6 (14)	1.01 (0.22–3.98)
Other household illnesses^*^	9 (39)	1 (3)	13.76 (2.77–137.17)
Nail biting	5 (22)	12 (27)	0.77 (0.23–2.38)
Thumb sucking^*^	5 (22)	12 (28)	0.75 (0.22–2.31)
Entered pen in kid barn	23 (100)	41 (93)	3.96 (0.36–541.80)
Fed does in kid barn	5 (22)	3 (7)	3.52 (0.85–16.66)
Visited doe barn^*^	12 (55)	20 (45)	1.42 (0.52–3.96)
Sat on hay bale at doe barn^*^	10 (45)	6 (14)	4.85 (1.54–16.40)
Used hand sanitizer after touching other animals (i.e., rabbits, dog)^*^	5 (26)	10 (25)	1.10 (0.31–3.61)
Used hand sanitizer after kid barn	15 (65)	19 (43)	2.39 (0.87–6.87)
Tasted samples	11 (48)	22 (50)	0.92 (0.34–2.49)

* *Variables with missing values: indicated variables had one (sex, thumb sucking, visited doe barn), two (age, sat on hay bale in doe barn), eight (used hand sanitizer after touching other animals), or 12 (other household illness) missing values. Exact logistic regression was used for variables with extremely small counts (<1) and median unbiased estimate is reported*.

**Table 2:** Multivariable analysis of risk factors for STEC O157 infection among children visiting farm X—Connecticut, 2016 (*n* = 63).

**Exposure category**	**Adjusted odds ratio (95% confidence interval)**
Age <5 years	1.70 (0.56–5.48)
Sat on hay bale at doe barn	4.55 (1.41–16.13)
Used hand sanitizer after kid barn	2.17 (0.73–6.81)

In 2016, the farm was open to the public during kidding season from March 5, 2016, until it was closed under the authority of the local public health officer on March 24, 2016, when the outbreak was identified. During weekends in March 2016, there were ~500 visitors per day. The farm produces cheeses, caramel candies, and milk that are sold on the farm or at local area stores. Visitors had access to the entirety of the farm, with the exception of the production building and milking parlor, and were allowed to enter the goat pens, with the exception of the buck pen and the pen in the doe barn. Visitors could sample or buy products at the farm store, and there were no restrictions on where food could be consumed. An interview with Farm X's owner and onsite visits by investigators indicated there were no public handwashing facilities for visitors to use before consuming samples or purchased food or after touching any of the animals. Hand sanitizer was available for visitors in the farm store and kid barn, but there were no signs encouraging visitors to use it. No hand sanitizer was available in the doe barn. The interview results indicated that bedding in the kid barn was not routinely changed; new bedding was piled on top of old. Bedding and birthing by-products were raked into pits in front of the pens until it was removed and added to an on-farm compost pile. Goat kids were also sold by Farm X as pets or for meat.

A total of 78 environmental and animal samples were collected from Farm X. Of these, 61 environmental samples were collected including swabs of the kid barn, doe barn, and farm store. Testing in the barns included the pens, barn floor, gutters, bedding, hay bales, rafters, troughs, walls inside pens, a grain holder, and muddy runoff from a compost pile. CT DoA collected 17 fecal samples and rectal swabs directly from kids and does to examine fecal shedding. Overall, 37% (29 of 78) of environmental and animal samples collected from Farm X and tested at the CT DPHL yielded STEC; of these, 62% (18 of 29) yielded the STEC O157 outbreak strain (Table 3).

**Table 3:** *E. coli* serogroup and pulsed-field gel electrophoresis (PFGE) pattern by isolation source during an illness outbreak linked to visiting farm X—Connecticut, 2016.

**STEC serogroup**	**Pattern**	**Patient samples (*****n*** **=** **50)**		**Goat samples (*****n*** **=** **17)**		**Environmental samples (*****n*** **=** **61)**	
		**No**.	**(%)**	**No**.	**(%)**	**No**.	**(%)**
O157	1	42^‡^	(76)	9^‡^	(53)	6	(8)
	2	2	(9)	0	(0)	0	(0)
	3	3	(7)	0	(0)	0	(0)
	4	1	(2)	0	(0)	0	(0)
	5	1	(2)	0	(0)	0	(0)
	6	1	(2)	0	(0)	1	(2)
	7	0	(0)	2	(0)	0	(3)
O103		2^‡^	(0)	7^‡^	(47)	5	(2)
O5		0	(0)	1	(13)	0	(8)

‡ *Two samples had both O157 and O103 isolated*.

The 18 samples yielding the outbreak strain of STEC O157 were isolated from samples collected from the kid and doe barns. In the kid barn, the outbreak strain was isolated from samples collected from the does and kids, bedding in the doe birthing and holding pens, a hay bale in a holding pen, the floor in front of a holding pen, and a rabbit cage. The outbreak strain was also isolated from three pooled samples collected from does in the doe barn where hay bales were lined against the fence so children could stand or sit on them to more easily feed or pet the does. PulseNet confirmed seven STEC O157 PFGE pattern combinations associated with this outbreak; of these, one was isolated from patients, goats, and the environment, one was isolated from patients and goats, four were isolated only from patients, and one was isolated exclusively from goats. STEC O103 was also isolated from goats, the environment, and a patient co-infected with the STEC O157 outbreak strain (Table 3).

**Figure 1:**
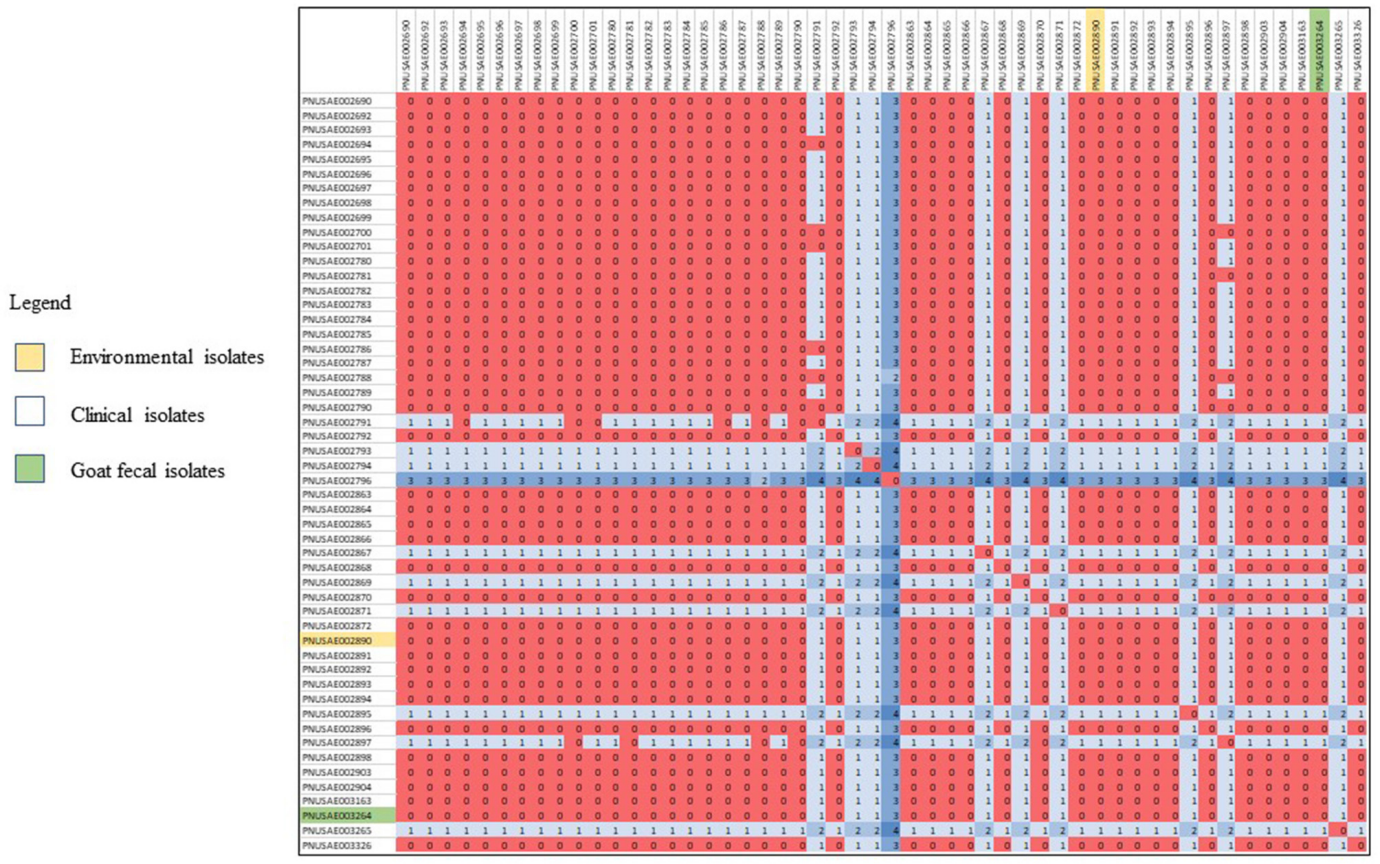
High-quality single nucleotide polymorphism (SNP) analysis of *E. coli* O157 sequences (*n* = 52) from isolates resulting from testing of clinical samples (*n* = 50), goat feces (*n* = 1), and an environmental sample from goat hay bedding (*n* = 1) during an outbreak of illness linked to a goat farm in Connecticut. Clinical and environmental isolates were highly related genetically within 0–4 STCPs. The high-quality SNPanalysis had phages masked, an internal reference PNUSAE002903-CT-M4158-160428_filtered_chromosomes.fasta, and was generated with Lyve-SET version 1.1.4f. Reads were cleaned with CG Pipeline (options: –no-singletons). SNPs were called with Varscan, and Lyve-SET was run with the following options: minimum coverage −20 min, alternative fraction −0.95, and allowed flanking −5 bp.

**Figure 2:**
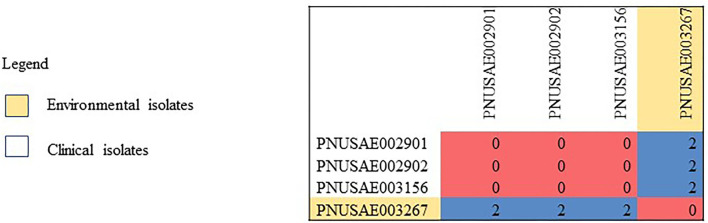
High-quality single nucleotide polymorphism (SNP) analysis of *E. coil* O103 sequences (*n* = 4) from isolates resulting from testing of clinical samples (*n* = 3) and environmental samples from goat hay bedding (*n* = 1) during an outbreak of illness linked to a goat farm in Connecticut. Clinical and environmental isolates were highly related genetically within 0–2 SNPs. The high-quality SNP analysis had phages masked, an internal reference PNUSAE003156-CT-M4158-160517_filtered_chromosomes.fasta, and was generated with Lyve-SET version 1.1.4f. Reads were cleaned with CG Pipeline (options: –no-singletons). SNPs were called with Varscan, and Lyve-SET was run with the following options: minimum coverage −20, min alternative fraction −0.95, and allowed flanking −5 bp.

Human, goat, and environmental isolates with the STEC O157 outbreak strain were highly related to one another by WGS high-quality single nucleotide polymorphism analysis (hqSNP) (Figure 1). The STEC O157 sequences from the outbreak clustered tightly within zero and four SNPs and the *E. coli* O103 sequences from the outbreak clustered within zero and two SNPs (Figures 1, 2). Public health actions were taken throughout the investigation. DPH provided public health messages through press releases including infection prevention through handwashing after contact with animals, after going to the bathroom, and by thoroughly cooking meats and washing fruits and vegetables. Farm X was closed to visitors by public health order on March 24th; the public health order was removed on April 13th. The CT DoA noted adequate milk pasteurization equipment and processes and testing of food samples did not yield STEC. The CT DoA also contacted Departments of Agriculture in states bordering CT where goat kids might have been sold as pets to residents, to inform them of the outbreak. Based on investigation findings, it was recommended that the farm owner provide public hand washing stations with soap, running water, and disposable towels for visitors to use when they exit animal areas. Owners and staff were advised to educate future farm visitors of the potential risks for transmission of diseases from animals or the environment. Additionally, it was recommended that Farm X restrict access to areas more likely to be contaminated with manure or birthing by-products, and not allow food, beverages, or strollers to enter any of the animal areas.

## Discussion

This investigation provided epidemiologic and laboratory evidence for an outbreak of STEC O157 infections resulting from exposure to goats and an animal environment that demonstrated widespread contamination caused by animals actively shedding *E. coli*. At the time of the investigation, this was Connecticut's largest zoonotic outbreak of STEC O157 infections recorded. Agritourism venue visitors might not be aware of the potential risk of illness from close contact with goats and other farm animals and might infrequently wash their hands after animal contact without appropriate guidance (25, 31, 32). Education for both the public who interact with farm animals as well as those who run these settings is essential (25). Contact with hay bales in the doe barn was associated with illness among children in this investigation. These hay bales were used to sit or stand on and abutted the pen of does which were shedding the outbreak strain of STEC O157. Greater than half of the patients were aged <5 years, and this investigation highlights the risks to young children from direct contact with goats and soiled bedding in the absence of infection prevention measures. This group is particularly at risk for acquiring infection due to factors such as hand-to-mouth habits, and it may be difficult for young children to access or use the hand hygiene stations at farms and petting zoos without assistance from an adult, to reach the hand sanitizer bottles on the tables, or the tap controls at the sink on their own (33–35). Restricting young children's direct contact to ruminants, such as preventing access inside animal pens and enclosures, may decrease risk to this vulnerable population (36). Since STEC O157 can be detected for >42 weeks in the environment, contact with the farm environment, even in the absence of direct contact with goats, may be an important risk factor for human illness (9, 37). Of note, three patients in this investigation did not have any identified epidemiologic link to the farm or goats from the farm but were infected with the predominant STEC O157 PFGE pattern combination found in this outbreak. All three of these patients reported onsets during March 18–25, which was during the height of the outbreak. One patient was a school-aged child who reported illness onset 8 days after another school-aged patient who was epidemiologically linked to the farm and from the same town. Another patient reported knowledge of Farm X and having a parent who worked near Farm X; however, neither patient nor parent visited the farm. The third patient reported pet therapy volunteer work at a health care facility. This may indicate transmission occurrence within the community.

This outbreak—the largest zoonotic STEC O157 outbreak in Connecticut—illustrates the risk to public health when recommendations outlined in the national guidelines for interacting with animals in public settings for cleaning, disinfection, and facility design are not followed. The absence of hand-washing stations for visitor use at Farm X and the limited availability of hand sanitizer may have contributed to the magnitude of this outbreak. Soap and clean running water should be used to wash hands, which should be dried with clean towels immediately upon exiting areas containing animals; alcohol-based hand sanitizers do not effectively inactivate *Cryptosporidium* which is a pathogen commonly carried by goats and other ruminants (13, 38). Reducing pathogen contamination in farm environments can be achieved through regular removal of soiled bedding from pens, avoiding piling fresh bedding on top of soiled bedding, ensuring that there is not run-off from manure piles into public areas, and ensuring that animals receive regular veterinary care (25, 36).

Limitations of this case-control study included reliance on self-reported data, which might have introduced social desirability and recall bias resulting in misclassification of exposures. This may lead to an overestimate of certain types of exposures, such as goat exposure or sitting on hay bales, and affect ability to detect differences between case-patients and controls. All case-patients and controls were children, so response bias might have occurred because parents were answering for their children. Controls self-selected by calling DPH after solicitation in a press release which might have introduced selection bias. A case-control study among adult farm visitors was not feasible as a result of the small number of cases among adults. Case-control studies might also be affected by confounding variables associated with observed risk factors.

Agritourism is growing in popularity, and working farms like the one involved in this outbreak are trying to connect with their communities through on-farm visits and animal contact. These connections are valuable for developing community relationships and can provide additional farm income. Other forms of agritourism which include contact with goats and kids, such as goat yoga, are also increasing in popularity. These activities likely present similar risks of human illness as described for this outbreak. In response to the outbreak investigation findings and the ongoing agritourism activities in the state, the CT DPH created an educational video to raise awareness of how to protect yourself around animals (39). The CT DoA held a 1-day conference for CT farmers and agritourism venue operators and discussed prevention measures and risk management for when they open to the public (40). Eliminating all risk from animal contact in agritourism settings might not be achievable. The public health goal is therefore to minimize the risk of disease and injury through facility design, animal husbandry, hand hygiene, and education.

## Data Availability Statement

The datasets presented in this study can be found in online repositories. The names of the repository/repositories and accession number(s) can be found at: https://www.ncbi.nlm.nih.gov/, PRJNA218110.

## Author Contributions

MN, PG, QP, KG-S, LG, JM, ML, PM, and MC conducted the field investigation, reviewed investigation tools, coordinated investigation activities with federal, state and local partners, and drafted, reviewed, and revised the manuscript. AB assisted with questionnaire development and revision, data analysis and drafted, reviewed, and revised the manuscript. MS, AMe, SO, EP, TN, LM, KH-T, AMc, DN, AMu, JR, NS, HM, and EV conducted the laboratory investigation, tested isolates, performed whole genome sequencing, analysis of sequencing data and drafted, reviewed, and revised the manuscript. MJL, WK, and BS collected samples from animals, led the on-farm animal component of the investigation and drafted, reviewed, and revised the manuscript. LW assisted with public communications for the investigation, reviewed press and media statements and drafted, reviewed, and revised the manuscript. All authors contributed to the article and approved the submitted version.

## Author Disclaimer

The conclusions and opinions in this report are those of the authors and do not necessarily represent the official position of the Centers for Disease Control and Prevention.

## Conflict of Interest

The authors declare that the research was conducted in the absence of any commercial or financial relationships that could be construed as a potential conflict of interest.

## Publisher's Note

All claims expressed in this article are solely those of the authors and do not necessarily represent those of their affiliated organizations, or those of the publisher, the editors and the reviewers. Any product that may be evaluated in this article, or claim that may be made by its manufacturer, is not guaranteed or endorsed by the publisher.
